# Identification and antimicrobial susceptibility profiles of *Nocardia* species clinically isolated in Japan

**DOI:** 10.1038/s41598-021-95870-2

**Published:** 2021-08-18

**Authors:** Masahiro Toyokawa, Noboru Ohana, Akiko Ueda, Minako Imai, Daiki Tanno, Mutsuko Honda, Yukiko Takano, Kazutaka Ohashi, Kyoichi Saito, Hiroki Shimura

**Affiliations:** 1 Preparing section for New Faculty of Medical Science, Fukushima Medical University, 1 Hikariga-oka, Fukushima City, 960-1295 Japan; 2 Department of Laboratory Medicine, Fukushima Medical University, Fukushima, Japan; 3 Laboratory for Clinical Investigation, Osaka University Hospital, Osaka, Japan; 4 Department of Clinical Laboratory Medicine, Fukushima Medical University Hospital, Fukushima, Japan

**Keywords:** Microbiology, Molecular biology

## Abstract

The aims of the present study were to profile the antimicrobial susceptibility patterns of a diverse range of *Nocardia* species isolated in Japan, and to determine the ability of matrix-assisted laser desorption ionization-time of flight mass spectrometry (MALDI-TOF MS) for species/complex identification. Identification of 153 clinical isolates was performed by full-length 16S rRNA gene sequencing as a reference method to evaluate the usefulness of MALDI-TOF MS identification. Antimicrobial susceptibility testing (AST) for 14 antibiotics was performed using the broth microdilution method against 146 of the isolates. Among the total 153 clinical isolates, *Nocardia farcinica* complex (25%) was the most common species, followed by *Nocardia cyriacigeorgica* (18%), *Nocardia brasiliensis* (9%), *Nocardia nova* (8%), and *Nocardia otitidiscaviarum* (7%). Among 150 isolates identified to the species/complex level by 16S rRNA gene sequencing, MALDI-TOF MS with the use of a supplemental *Nocardia* library (JMLD library ver.ML01) correctly identified 97.3% (n = 146) to the species/complex level and 1.3% (n = 2) to the genus level. Among the 146 *Nocardia* isolates that underwent AST, the susceptibilities were 100% to linezolid, 96% to amikacin, 94% to trimethoprim-sulfamethoxazole, and 76% to imipenem. None of the trimethoprim-sulfamethoxazole-resistant isolates carried either plasmid-mediated sulfonamide-resistant genes (*sul1*, *sul2*) or trimethoprim-resistant genes (*dfrA*).

## Introduction

*Nocardia* species are ubiquitous environmental organisms that can cause local or disseminated infection in humans. The lung is the most common primary site of infection, and central nervous system infection is often encountered through hematogenous dissemination from a pulmonary focus, particularly in immunocompromised hosts^1^. Prompt diagnosis and appropriate treatment of nocardiosis are required, because it is a fatal infection^2^. Trimethoprim-sulfamethoxazole (TMP-SMX) is the first-line agent for initial therapy for nocardiosis^3^, thus accurate determination of the susceptibility to TMP-SMX in clinical isolates is crucial. Conville et al. reported that the standard broth microdilution method for TMP-SMX may cause false resistance because of difficulties in end-point interpretation, and they recommend performing a confirmation test via sulfisoxazole disk diffusion testing^4^. Unfortunately we are unable to purchase sulfisoxazole disks in Japan; therefore there is no reliable data concerning *Nocardia* isolates resistant to TMP-SMX in Japan. On the other hands, identification of clinical isolates to the species/complex level is important, because *Nocardia* species differ in clinical spectrum and susceptibility patterns^5–7^. Gene sequencing such as 16S rRNA gene is currently used for the identification of *Nocardia* species; however, recently, matrix-assisted laser desorption ionization-time of flight mass spectrometry (MALDI-TOF MS)-based identification has been identified as a rapid, easy and reliable method^8,9^.

The aims of the present study were to profile the antimicrobial susceptibility patterns of a diverse range of *Nocardia* species isolated from clinical specimens in Japan, and to determine the utility of MALDI-TOF MS for the routine identification of *Nocardia* species.

## Materials and methods

### Bacterial isolates and identification

A total of 153 clinical isolates of *Nocardia* species recovered from patients in 25 microbiology laboratories in Japan were studied. The isolates were cultured from respiratory tract specimens (n = 90), skin and soft tissue (n = 14), blood (n = 5), deep abscess (n = 6), pleural effusion (n = 1), ascitic fluid (n = 1), synovial fluid (n = 1), others (n = 5) and unknown (n = 30). Identification of *Nocardia* species was based on Gram stain, colonial morphology and molecular technique. All isolates were identified by full-length 16S rRNA gene sequencing, for which the universal primers 8UA (5′-AGAGTTTGATCMTGGCTCAG-3′) and 1485B (5′-ACGGGCGGTGTGTRC-3′) were used, as described previously^10^. We performed sequencing analysis using a GenBank BLAST search and EzBioCloud (https://www.ezbiocloud.net/identify/result?id=5ef99cb3c39ad461094e9aa3). Previously established criteria for identification of *Nocardia* isolates to the species or complex level were followed^11,12^.

### MALDI-TOF MS identification

All *Nocardia* species isolates were analyzed using a Microflex LT bench top mass spectrometer (Bruker Daltonics, Germany). MALDI Biotyper 3.1 software (Bruker Daltonics, MALDI Biotyper reference library version 8.0.0.0) was applied with the use of a supplemental *Nocardia* library (JMLD library ver.ML01, containing 114 Main Spectras for 46 *Nocardia* species) provided by BCKK MALDINOMICS (Beckman Coulter Japan, Tokyo, Japan). The isolates were cultivated on 5% sheep blood agar plates at 35℃, and tested at 18 and 48 h, an early stage of growth^8^. Samples were prepared as previously described (on-plate extraction)^8^. Protein extraction was also performed using the formic acid/ethanol method according to the Bruker Daltonics’ protocol for any isolate failed to be identified by on-plate extraction. A spectral score of ≥ 2.00 was considered identification to the species level, a score of 1.700–1.999 indicated identification at the genus level, and a score of < 1.70 was considered unreliable identification.

Complex level identification was performed on some *Nocardia* species according to Conville’s criteria^11^.

*Nocardia asteroides* ATCC 23206, *Nocardia brasiliensis* ATCC 23238, *Nocardia farcinica* ATCC 23157, and *Nocardia otitidiscaviarum* ATCC 23240 were used as the quality control strains.

### Antimicrobial susceptibility testing (AST)

AST was performed using the broth microdilution method with frozen panels (Eiken Chemical, Tokyo, Japan), according to the Clinical and Laboratory Standards Institute (CLSI) M24-A2 guidelines^13^ against 146 clinical isolates. In brief, a heavy organism suspension was prepared in a small volume of sterile saline with 7–10 3-mm glass beads and was vortexed vigorously. Clumps were allowed to settle for 15 min, and the supernatant was adjusted to a 0.5 McFarland standard using a calibrated nephelometer. For frozen panel inoculation, the adjusted 0.5 McFarland suspension was diluted 30-fold with sterile saline and 10 µl of the diluted solution was dispensed into each well of the panel. The panels were incubated at 35℃ for 72 h until moderate growth was observed in the growth control wells. For TMP-SMX, the MICs were determined as the wells corresponding to 80% inhibition of growth compared to the controls. The MICs were determined for TMP-SMX, amikacin, tobramycin, ceftriaxone, imipenem, minocycline, linezolid, ciprofloxacin, moxifloxacin, clarithromycin, cefotaxime (100 isolates only), meropenem (100 isolates only), tigecycline (100 isolates only), and arbekacin (100 isolates only), and interpreted as recommended by CLSI. *Staphylococcus aureus* ATCC 29213, *Pseudomonas aeruginosa* ATCC 27853 and *Enterococcus faecalis* ATCC 29212 were used as the quality control strains.

For confirmation of TMP-SMX resistance, disk diffusion testing with a 250-μg sulfisoxazole disk (Hardy Diagnostics, CA, USA)^4^ was performed in all 21 TMP-SMX-resistant isolates determined by AST (MIC ≥ 4/76 μg/ml). Moreovere, re-analysis of the broth microdilution method using panels with different lots and inoculum colony count were also performed. For this analysis, *Nocardia nova* ATCC BAA-2227 and *Escherichia coli* ATCC 25922 were used as the quality control strains.

### Detection of plasmid-mediated TMP-SMX-resistant genes

The plasmid-mediated sulfonamide-resistant genes (*sul1*, *sul2*)^14^ and trimethoprim-resistant gene (*dfrA*)^15^ were detected by PCR in 21 TMP-SMX-resistant isolates, determined by AST (MIC ≥ 4/76 μg/ml) (see Table S1 for the primer sequence and Figure S1 for a gel electrophoresis image of positive controls in the supplemental material).

The present study was conducted in accordance with the ethical guidelines of the Ministry of Health, Labor and Welfare of Japan. No ethical committee approvals or informed consent were needed for this study.

### Conference presentation

A part of this study was presented at the 27th European Congress of Clinical Microbiology and Infectious Diseases, 22 to 25 April 2017, Vienna, Austria [abstr. #316].

## Results

### Identification of clinical isolates by full-length 16S rRNA gene sequencing

Among the 153 clinical isolates, 150 were identified to the species/complex level, including 24 different species/complexes, and the remaining three isolates were identified to the genus level (Table 1). *Nocardia farcinica* complex (n = 39; 25%) was the most common species, followed by *Nocardia cyriacigeorgica* (n = 27; 18%), *Nocardia brasiliensis* (n = 14; 9%), *Nocardia nova* (n = 12; 8%), *Nocardia otitidiscaviarum* (n = 11; 7%), *Nocardia elegans* (n = 10; 7%), *Nocardia beijingensis* (n = 7; 5%), *N. nova* complex (n = 4), *Nocardia abscessus* (n = 4), *Nocardia asiatica* (n = 4), *Nocardia wallacei* (n = 3), *Nocardia* sp. (n = 3), *Nocardia transvalensis* complex (n = 3), *N. abscessus* complex (n = 2), *Nocardia thailandica* (n = 2), and one each of *Nocardia aobensis*, *Nocardia arthritidis*, *Nocardia asteroides*, *Nocardia takedensis*, *Nocardia pseudobrasiliensis*, *Nocardia yamanashiensis*, *Nocardia mexicana*, and *Nocardia vinacea*.

**Table 1:** Comparison of full-length 16SrRNA gene sequencing and MALDI-TOF MS identification for 153 *Nocardia* spp. isolates using the manufactuer's library combined with a custom library.

16SrRNA gene sequencing identification (no. of isolates tested)	MALDI-TOF MS identification	No. of isolates identified by MALDI-TOF MS at a cutoff score level of:			
		≥ 2.0	≥ 1.7	< 1.7(no identification)	No. of isolated misidentification
*N. farcinica* complex (n = 39)	*N. farcinica* complex	39	0	0	0
*N. cyriacigeorgica* (n = 27)	*N. cyriacigeorgica* complex	27	0	0	0
*N. brasiliensis* (n = 14)	*N. brasilensis*	14	0	0	0
*N. otitidiscaviarum* (n = 11)	*N. otitidiscaviarum* complex	11	0	0	0
*N. nova* (n = 12)	*N. nova* complex	12	0	0	0
*N. elegans* (n = 10)		10	0	0	0
*N. aobensis* (n = 1)		1	0	0	0
*N. nova* complex (n = 4)		4	0	0	0
*N. abcessus* (n = 4)	*N. abscessus* complex	4	0	0	0
*N. asiatica* (n = 4)		4	0	0	0
*N. beijingensis* (n = 7)		5	2^b^	0	0
*N. arthritidis* (n = 1)		1	0	0	0
*N. abcessus* complex (n = 2)		1	0	1	0
*N. wallacei* (n = 3)	*N. transvalensis* complex	3	0	0	0
*N. transvalensis* complex (n = 3)		2	0	0	1^c^
*N. thailandica* (n = 2)	*N. thailandica*	2	0	0	0
*N. asteroides* (n = 1)	*N. asteroides*	1	0	0	0
*N. takedensis* (n = 1)	*N. takedensis*	1	0	0	0
*N. pseudobrasiliensis* (n = 1)	*N. pseudobrasiliensis*	1	0	0	0
*N. yamanashiensis* (n = 1)	*N. yamanashiensis*	1	0	0	0
*N. mexicana* (n = 1)	*N. mexicana*	1	0	0	0
*N. vinacea* (n = 1)	*N. vinacea*	1	0	0	0
All 150 isolates (%)		146 (97.3)	2 (1.3)	1 (0.7)	1 (0.7)
*Nocardia* sp. (n = 3)^a^	*N. testacea*	3	0	0	0

^a^2/3 isolates were identified as *N. testacea*/*N. sienata*, and 1/3 isolates was identified as *N. testacea*/*N. flavorosea*/*N. sienata*/*N.rhamnosiphila*. ^b^Two isolates were identified as *N. araoensis* with scores of 1.91 and 1.99, respectively. ^c^misidentified as *N. brasilliensis* with a score of 2.033.

**Figure 1:**
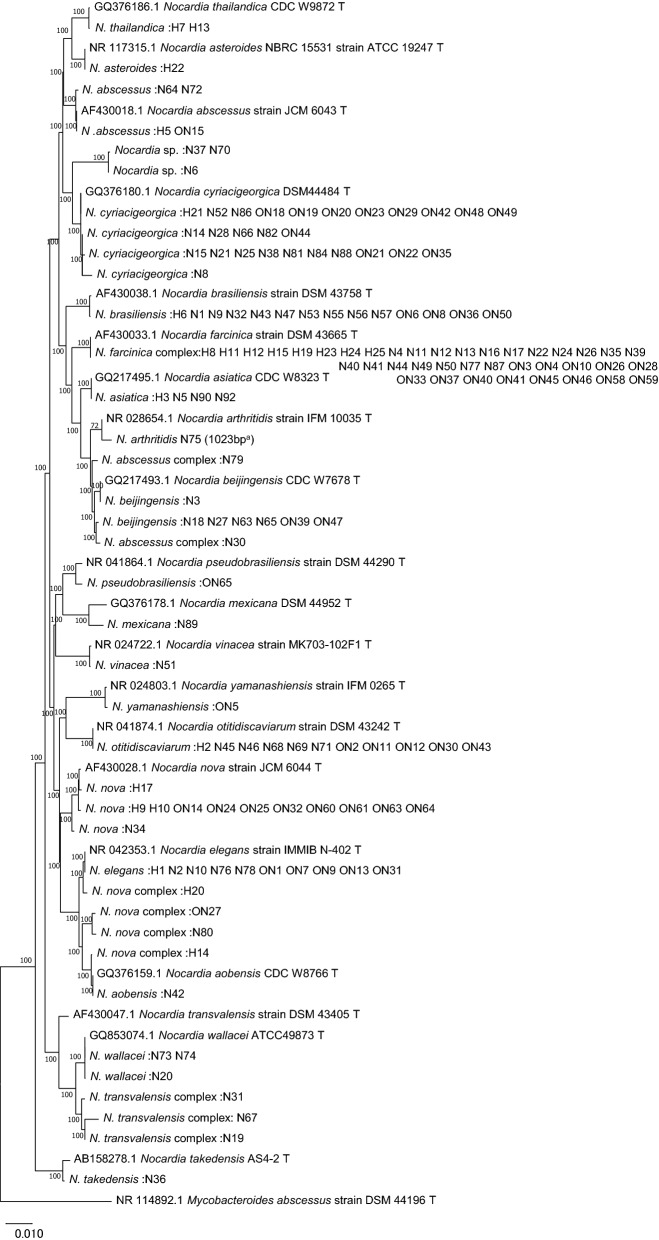
16S rRNA sequence-based phylogenetic tree of clinical isolates of *Nocardia* with those of closely related species. The evolutionary history was inferred using the Neighbor-Joining method^16^. The optimal tree is shown. The tree is drawn to scale, with branch lengths in the same units as those of the evolutionary distances used to infer the phylogenetic tree. The evolutionary distances were computed using the Maximum Composite Likelihood method^17^ and are in the units of the number of base substitutions per site. The proportion of sites where at least 1 unambiguous base is present in at least 1 sequence for each descendent clade is shown next to each internal node in the tree. This analysis involved 59 nucleotide sequences. Codon positions included were 1st + 2nd + 3rd + Noncoding. All ambiguous positions were removed for each sequence pair (pairwise deletion option). There were a total of 1405 positions in the final dataset. Evolutionary analyses were conducted in MEGA X^18^. ^a^ The read length of N75 strain was 1023 bp with a good quality sequence.

*N. cyriacigeorgica* was the most frequently isolated *Nocardia* species from the respiratory tract (28%; 25/90), followed by *N. farcinica* complex (21%; 19/90). *N. brasiliensis* was isolated in one-half (50%; 7/14) of the skin and soft tissue samples. Figure 1 shows the alignment of 1405 bases of the 16SrRNA gene of all clinical isolates of *Nocardia* with those of closely related species obtained using the neighbor-joining method^16,17^ with MEGA X software^18^.

### MALDI-TOF MS identification

Among the 150 isolates that had identified to the species/complex level by 16S rRNA gene sequencing, MALDI-TOF MS correctly identified 97.3% (146/150) to the species/complex level and 1.3% (2/150) to the genus level (Table 1). One isolate, which was identified as *N. transvalensis* complex by 16S rRNA gene sequencing, was misidentified as *N. brasiliensis,* with a score ≥ 2.00. In addition, there was one isolate that could not be identified, with a score of < 1.70. All three isolates identified to the genus level by 16S rRNA gene sequencing were identified as *Nocardia testacea*, with scores of ≥ 2.00.

### Antimicrobial susceptibility testing

The MIC range, MIC_50_, MIC_90_ and susceptibility for the seven most frequently isolated *Nocardia* species/complexes are shown in Table 2. The antibiograms of the uncommon *Nocardia* species are listed in Table 3.

**Table 2:** Activities of antimicrobial agents against the 7 most frequently isolated *Nocardia* species/complexes.

Bacterium (no of isolates tested) and antimicrobial agent	MIC (μg/ml)			Susceptibility (%)^a^		
	Range	50%	90%	S	I	R
*N. farcinica* complex^b^ (37)						
Amikacin	0.06–4	1	2	37 (100)	0 (0)	0 (0)
Tobramycin	≤ 0.015–> 64	32	64	1 (3)	1 (3)	35 (94)
Arbekacin (26)	0.03–2	0.5	1	–	–	–
Trimethoprim-sulfamethoxazole	0.25/4.75–4/76	2/38	4/76	24 (65)	–	13 (35)
Ceftriaxone	1–> 128	64	256	1 (3)	10 (27)	26 (70)
Cefotaxime (26)	0.125–> 256	64	> 256	1 (4)	4 (15)	21 (81)
Imipenem	0.5–8	2	4	35 (95)	2 (5)	0 (0)
Meropenem (26)	0.5–16	4	16	–	–	–
Linezolid	0.5–4	4	4	37 (100)	0 (0)	0 (0)
Ciprofloxacin	0.25–16	2	8	17 (46)	4 (11)	16 (43)
Moxifloxacin	≤ 0.015–8	1	2	23 (62)	12 (32)	2 (5)
Clarithromycin	0.5–> 64	> 64	> 64	1 (3)	0 (0)	36 (97)
Minocycline	0.06–4	4	4	4 (11)	33 (89)	0 (0)
Tigecycline (26)	0.25–> 16	16	> 16	–	–	–
*N. cyriacigeorgica* (27)						
Amikacin	0.125–4	0.5	2	27 (100)	0 (0)	0 (0)
Tobramycin	0.125–1	0.25	0.5	27 (100)	0 (0)	0 (0)
Arbekacin (15)	0.5–2	0.5	2	–	–	–
Trimethoprim-sulfamethoxazole	0.25/4.75–4/76	0.5	2/38	26 (96)	–	1 (4)
Ceftriaxone	1–16	4	16	23 (85)	4 (15)	0 (0)
Cefotaxime (15)	2–32	4	16	11 (73)	4 (27)	0 (0)
Imipenem	0.25–8	2	4	26 (96)	1 (4)	0 (0)
Meropenem (15)	2–8	4	8	–	–	–
Linezolid	2–4	4	4	27 (100)	0 (0)	0 (0)
Ciprofloxacin	4–32	16	32	0 (0)	0 (0)	27 (100)
Moxifloxacin	1–8	4	8	1 (4)	8 (30)	18 (66)
Clarithromycin	8–> 64	> 64	> 64	0 (0)	0 (0)	27 (100)
Minocycline	2–8	2	4	0 (0)	27 (100)	0 (0)
Tigecycline (15)	2–16	8	16	–	–	–
*N. nova* complex^c^ (23)						
Amikacin	0.06–1	0.25	0.5	23 (100)	0 (0)	0 (0)
Tobramycin	0.25–> 256	64	256	3 (13)	1 (4)	19 (83)
Arbekacin (13)	0.125–0.5	0.25	0.5	–	–	–
Trimethoprim-sulfamethoxazole	0.125/2.375–2/38	1/19	2/38	23 (100)	0 (0)	0 (0)
Ceftriaxone	≤ 0.06–16	8	16	18 (78)	5 (22)	0 (0)
Cefotaxime (13)	4–32	8	16	8 (62)	5 (38)	0 (0)
Imipenem	≤ 0.015–0.25	0.125	0.25	23 (100)	0 (0)	0 (0)
Meropenem (13)	0.125–1	0.5	1	–	–	–
Linezolid	≤ 0.25–4	2	4	23 (100)	0 (0)	0 (0)
Ciprofloxacin	4–32	8	16	0 (0)	0 (0)	23 (100)
Moxifloxacin	2–> 4	2	> 4	0 (0)	12 (52)	11 (48)
Clarithromycin	0.03–> 64	0.06	0.25	22 (96)	0 (0)	1 (4)
Minocycline	≤ 0.5–8	4	8	1 (4)	19 (83)	3 (13)
Tigecycline (13)	4–> 16	16	> 16	–	–	–
*N. abscessus* complex^d^ (18)						
Amikacin	0.125–0.25	0.25	0.25	18 (100)	0 (0)	0 (0)
Tobramycin	0.25–1	0.5	1	18 (100)	0 (0)	0 (0)
Arbekacin (15)	0.03–0.125	0.06	0.125	–	–	–
Trimethoprim-sulfamethoxazole	0.25/4.75–2/38	0.5/9.5	1/19	18 (100)	0 (0)	0 (0)
Ceftriaxone	0.5–16	2	16	16 (89)	2 (11)	0 (0)
Cefotaxime (15)	0.5–16	4	8	14 (93)	1 (7)	0 (0)
Imipenem	0.25–32	2	32	13 (72)	2 (11)	3 (17)
Meropenem (15)	0.5–4	1	4	–	–	–
Linezolid	0.25–4	2	4	18 (100)	0 (0)	0 (0)
Ciprofloxacin	0.5–> 32	8	> 32	4 (22)	1 (6)	13 (72)
Moxifloxacin	0.125–> 32	2	> 32	3 (17)	6 (33)	9 (50)
Clarithromycin	0.25–> 64	16	> 64	4 (22)	2 (11)	12 (67)
Minocycline	≤ 0.5–4	1	4	12 (67)	6 (33)	0 (0)
Tigecycline (15)	0.5–> 16	1	> 16	–	–	–
*N. brasiliensis* (14)						
Amikacin	0.25–4	2	4	14 (100)	0 (0)	0 (0)
Tobramycin	0.125–0.5	0.25	0.25	14 (100)	0 (0)	0 (0)
Arbekacin (10)	0.5–1	0.5	1	–	–	–
Trimethoprim-sulfamethoxazole	0.25/4.75–1/19	0.5/9.5	0.5/9.5	14 (100)	0 (0)	0 (0)
Ceftriaxone	2–> 256	64	> 256	3 (21)	3 (21)	8 (57)
Cefotaxime (10)	16–> 256	> 256	> 256	0 (0)	2 (20)	8 (80)
Imipenem	1–> 32	> 32	> 32	2 (14)	0 (0)	12 (86)
Meropenem (10)	4–8	8	8	–	–	–
Linezolid	4–8	4	8	14 (100)	0 (0)	0 (0)
Ciprofloxacin	2–8	8	8	0 (0)	0 (0)	14 (100)
Moxifloxacin	0.5–2	1	2	11 (79)	3 (21)	0 (0)
Clarithromycin	8–> 64	> 64	> 64	0 (0)	0 (0)	14 (100)
Minocycline	1–4	2	4	2 (14)	12 (86)	0 (0)
Tigecycline (10)	1–4	1	2	–	–	–
*N. otitidiscaviarum* (11)						
Amikacin	0.5–2	1	2	11 (100)	0 (0)	0 (0)
Tobramycin	1–64	4	16	6 (55)	3 (27)	2 (18)
Arbekacin (6)	0.125–0.25	0.125	0.25	–	–	–
Trimethoprim-sulfamethoxazole	0.5/9.5–4/76	1/19	4/76	8 (73)	–	3 (27)
Ceftriaxone	128–> 256	> 256	> 256	0 (0)	0 (0)	11 (100)
Cefotaxime (6)	> 256	> 256	> 256	0 (0)	0 (0)	6 (100)
Imipenem	8–> 32	> 32	> 32	0 (0)	0 (0)	11 (100)
Meropenem (6)	8–> 32	16	> 32	–	–	–
Linezolid	1–8	4	4	11 (100)	0 (0)	0 (0)
Ciprofloxacin	2–32	4	16	0 (0)	3 (27)	8 (73)
Moxifloxacin	1–8	2	8	2 (18)	4 (36)	5 (45)
Clarithromycin	> 64	> 64	> 64	0 (0)	0 (0)	11 (100)
Minocycline	0.125–2	0.5	1	10 (91)	1 (9)	0 (0)
Tigecycline (6)	0.5–2	1	2	–	–	–
*N. transvalensis* complex^e^ (6)						
Amikacin	16–> 256	128	> 256	0 (0)	0 (0)	6 (100)
Tobramycin	> 256	> 256	> 2 56	0 (0)	0 (0)	6 (100)
Arbekacin	4–16	4	16	–	–	–
Trimethoprim-sulfamethoxazole	0.25/4.75–8/152	1/19	8/152	4 (67)	–	2 (33)
Ceftriaxone	0.5–16	1	16	5 (83)	1 (17)	0 (0)
Cefotaxime	0.5–32	2	32	5 (83)	1 (17)	0 (0)
Imipenem	0.5–> 32	4	> 32	3 (50)	1 (17)	2 (33)
Meropenem	0.125–8	0.5	8	–	–	–
Linezolid	1–4	2	4	6 (100)	0 (0)	0 (0)
Ciprofloxacin	0.5–4	1	4	5 (83)	0 (0)	1 (17)
Moxifloxacin	0.125–32	0.25	32	5 (83)	0 (0)	1 (17)
Clarithromycin	2–64	8	64	1 (17)	0 (0)	5 (83)
Minocycline	1–4	2	4	1 (17)	5 (83)	0 (0)
Tigecycline	4–> 16	16	> 16	–	–	–

^a^S, susceptible; I, intermediate; R, resistant. ^b^*N. farcinica* and *Nocardia kroppenstedtii* were included in the *N. farcinica* complex. ^c^*N. nova*, *N. elegans*, and *N. aobensis* were included in the *N. nova* complex. ^d^*N. abscessus*, *N. asiatica*, *N. beijingensis*, and *N. arthritidis* were included in the *N. abscessus* complex. ^e^*N. wallacei* and *N. transvalensis* were included in the *N. transvalensis* complex.

**Table 3:** Activities of antimicrobial agents against 10 clinical isolates of uncommon *Nocardia* species.

Antimicrobial agent	MIC (μg/ml)^a^ for						
	*Nocardia* sp. (3)^b^	*N. thailandica* (2)	*N. asteroides* (1)	*N. takedensis* (1)	*N. yamanashiensis* (1)	*N. mexicana* (1)	*N. vinacea* (1)
Amikacin	0.06–0.125 (100)	0.5–1 (100)	0.25 (S)	0.25 (S)	0.25 (S)	8 (S)	0.25 (S)
Tobramycin	0.03 (100)	0.06–8 (50)	0.06 (S)	1 (S)	4 (S)	> 256 (R)	0.06 (S)
Arbekacin	≤ 0.015	0.125	0.125	0.125	–	1	0.03
Trimethoprim-sulfamethoxazole	2/38 (100)	2/38–4/76 (50)	0.5/9.5 (S)	0.125/2.375 (S)	0.25/4.75 (S)	4/76 (R)	0.25/4.75 (S)
Ceftriaxone	8 (100)	8–> 256 (50)	16 (I)	2 (S)	2 (S)	2 (S)	0.25 (S)
Cefotaxime	8–16 (33)	16–> 256 (0)	32 (I)	4 (S)	–	4 (S)	0.5 (S)
Imipenem	0.5–1 (100)	1–32 (50)	1 (S)	0.5 (S)	1 (S)	1 (S)	0.5 (S)
Meropenem	2	2–8	1	1	–	0.5	0.5
Linezolid	2 (100)	4 (100)	4 (S)	2 (S)	1 (S)	4 (S)	2 (S)
Ciprofloxacin	0.125–0.25 (100)	16–32 (0)	16 (R)	4 (R)	1 (S)	> 32 (R)	2 (I)
Moxifloxacin	0.125–0.25 (100)	2 (0)	4 (R)	2 (I)	0.25 (S)	16 (R)	1 (S)
Clarithromycin	> 64 (R)	0.125–> 64 (50)	32 (R)	4 (I)	0.5 (S)	32 (R)	0.25 (S)
Minocycline	4 (0)	1 (100)	2 (I)	1 (S)	1 (S)	4 (I)	1 (S)
Tigecycline	1–2	0.5–1	4	4	–	> 16	0.125

^a^The MIC range is shown, with the percentage of susceptible isolates or susceptibility category (S, susceptible; I, intermediate; R, resistant) in parentheses. The number of isolates for each species is shown in parentheses. ^b^2/3 isolates were identified as *N. testacea*/*N. sienata*, and 1/3 isolates was identified as *N. testacea*/*N. flavorosea*/*N. sienata*/*N.rhamnosiphila* by full-length 16S rRNA gene sequencing.

Overall, linezolid was the most active drug across all species, with no in vitro resistance. Among the 146 *Nocardia* isolates that underwent AST, 96% were susceptible to amikacin; 86% were susceptible to TMP-SMX; and 76% were susceptible to imipenem. In contrast, about 80% of the *Nocardia* isolates were not susceptible to clarithromycin, minocycline or ciprofloxacin. Six amikacin-resistant isolates were *N. transvalensis* complex. The results of disk diffusion testing with a 250-μg sulfisoxazole disk and re-analysis of the broth microdilution method against 21 TMP-SMX-resistant isolates are shown in Table 4. Of these 21 isolates, five were interpreted as being TMP-SMX-resistant, and 12 were susceptible, while four were not interpretable. Finally, 94% (137/146) of the *Nocardia* isolates were determined to be susceptible to TMP-SMX. The isolates that were not susceptible to TMP-SMX (including isolates not interpretable) were found among *N. otitidiscaviarum* (27%; 3/11), *N. farcinica* complex (8%; 3/37), *N. cyriacigeorgica* (4%; 1/27), *N. thailandica* (1/2) and *N. mexicana* (1/1).

**Table 4:** Results of disk diffusion testing with a 250-μg sulfisoxazole disk and re-analysis of broth microdilution method against 21 TMP-SMX-resistant isolates.

Bacterium	First mesurement	Re-analysis		Zone diameter (mm) ^a^ around the 250-μg sulfisoxazole disk (duplicate)
	MIC (μg/ml) of trimethoprim-sulfamethoxazole	MIC (μg/ml) of trimethoprim-sulfamethoxazole (duplicate)	Colony count; Ideal inoculum consentration (1 × 10^5^ to 5 × 10^5^ CFU/ml)	
*N. cyriacigeorgica*	4	4, 4	2.1 × 10^5^	≤ 15, ≤ 15
*N. farcinica* complex	4	2, 2	2.8 × 10^5^	≥ 35, ≥ 35
*N. farcinica* complex	4	2, 2	1.7 × 10^5^	≥ 35, ≥ 35
*N. farcinica* complex	4	2, 2	1.7 × 10^5^	30, 30
*N. farcinica* complex	4	4, 2	1.1 × 10^5^	≥ 35, ≥ 35
*N. farcinica* complex	4	4, 4	1.7 × 10^5^	32, 31
*N. farcinica* complex	4	2, 2	1.8 × 10^5^	≥ 35, ≥ 35
*N. farcinica* complex	4	4, 2	1.4 × 10^5^	≥ 35, ≥ 35
*N. farcinica* complex	4	4, 4	1.5 × 10^5^	≥ 35, ≥ 35
*N. farcinica* complex	4	4, 2	1.8 × 10^5^	25, 25
*N. farcinica* complex	4	4, 2	3.2 × 10^5^	≥ 35, ≥ 35
*N. farcinica* complex	4	8, 4	2.5 × 10^5^	≥ 35, ≥ 35
*N. farcinica* complex	4	4, 4	2.3 × 10^5^	≥ 35, ≥ 35
*N. farcinica* complex	4	4, 2	1.5 × 10^5^	≥ 35, ≥ 35
*N. mexicana*	4	8, 4	< 1 × 10^5^	≤ 15, ≤ 15
*N. otitidiscaviarum*	4	4, 4	< 1 × 10^5^	≤ 15, ≤ 15
*N. otitidiscaviarum*	4	4, 4	< 1 × 10^5^	≤ 15, ≤ 15
*N. otitidiscaviarum*	4	4, 4	1.2 × 10^5^	≤ 15, ≤ 15
*N. thailandica*	4	4, 2	1.6 × 10^5^	30, 30
*N. transvalensis* complex	8	> 8, 8	1.4 × 10^5^	≥ 35, ≥ 35
*N. transvalensis* complex	8	> 8, > 8	1.3 × 10^5^	≥ 35, ≥ 35

^a^ A zone ≥ 35 mm indicates susceptibility, zones between 16 and 34 mm are not interpretable, and a zone ≤ 15 mm indicates resistance.

Susceptibility varied according to *Nocardia* species. Among the frequently isolated species, nonsusceptibility to imipenem was high in *N. otitidiscaviarum* (100%) and *N. brasiliensis* (86%), while that was low in *N. nova* complex (0%), *N. cyriacigeorgica* (4%) and *N. farcinica* complex (5%). More than 80% susceptibility to ceftriaxone was shown in *N. abscessus* complex (89%), *N. cyriacigeorgica* (85%) and *N. transvalensis* complex (83%). *N. nova* complex showed a good susceptibility to clarithromycin, although its resistance rate in other frequently isolated species was high. Susceptibility to fluoroquinolones including moxifloxacin was low among all the *Nocardia* species except *N. transvalensis* complex and *N. brasiliensis.*

For the 100 *Nocardia* isolates, the MIC_50_ and MIC_90_ values of tigecycline were 8 and > 16 μg/ml, respectively. These values for *N. brasiliensis* and *N. otitidiscaviarum*, 1 and 2 μg/ml, respectively, were lower than those for the other frequently isolated *Nocardia* species. The MIC_50_ and MIC_90_ values of minocycline for those 100 *Nocardia* isolates were 4 and 4 μg/ml, respectively.

**Figure 2:**
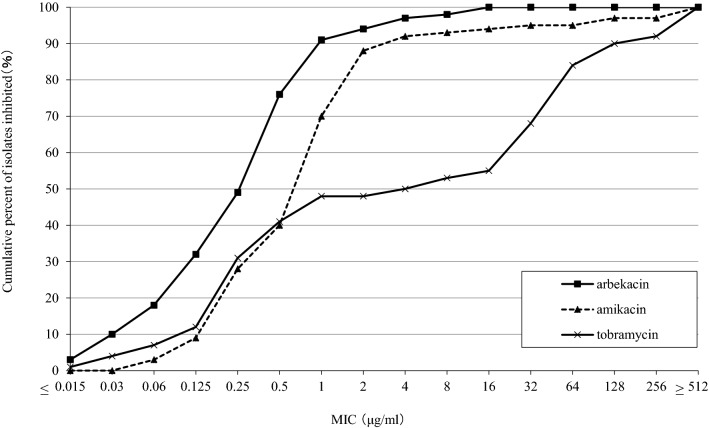
MIC distributions of arbekacin, amikacin and tobramycin against 100 *Nocardia* isolates.

The cumulative percentages of the 100 *Nocardia* isolates inhibited by each concentration of arbekacin, amikacin and tobramycin are shown in Fig. 2. The MIC_50_ and MIC_90_ values of arbekacin, amikacin and tobramycin were 0.25 and 1, 1 and 4, and 4 and 128 μg/ml, respectively. Arbekacin showed low MIC values (4–16 μg/ml) even against *N. transvalensis* complex, which included high-level amikacin-resistant isolates (> 256 μg/ml).

### Detection of plasmid-mediated TMP-SMX-resistant genes

None of the five TMP-SMX-resistant isolates or the remaining 16 isolates (TMP-SMX ≥ 4/76 μg/ml) carried either plasmid-mediated sulfonamide-resistant genes (*sul1*, *sul2*) or trimethoprim-resistant gene (*dfrA*).

## Discussion

There is limited information about the distribution and antimicrobial susceptibility of various *Nocardia* species in Japan. In the present study, *N. farcinica* complex (25%) was the most common species, followed by *N. cyriacigeorgica* (18%), *N. brasiliensis* (9%), *N. nova* (8%), and *N. otitidiscaviarum* (7%), according to full-length 16S rRNA gene sequence identification. When using the complex criteria for MALDI-TOF MS identification^11^, *N. farcinica* complex (25%) remained the most predominant, but the next most dominant species were *N. cyriacigeorgica* and *N. nova* complex (18.3% each), followed by the *N. abscessus* complex (12%), and *N. brasiliensis* (9%). These epidemiological data, taken together with the antimicrobial susceptibility profiles of different species/complexes, may contribute to accurate empirical treatment decisions.

The current study demonstrates that MALDI-TOF MS is useful for rapidly and accurately providing species/complex identification of *Nocardia* species. The direct spotting and standard bacterial extraction methods developed for MALDI-TOF MS are suboptimal for *Nocardia* species, due to the hardness and composition of the cell wall^7^. Previous studies have stressed the need for enhanced sample preparation methods to sufficiently identify *Nocardia* species^9,19–21^. Khot et al. reported that the age of *Nocardia* cultures plays an important role in the success of MALDI-TOF MS identification, and recommended to use a colony at an early stage of growth^8^. Their method correctly identified 82.8% (72/87) to the species/complex level and 11.5% (10/87) to the genus level if the cut-off for species-level identification was lowered from a score of ≥ 2.00 to ≥ 1.90. The results of the current study indicate that our method is more reliable than Khot’s method, despite the strict threshold value for species-level identification being used. The point of the method used in the present study was to use a colony at an early stage of growth (18–48 h cultivation), and to use a considerably augmented reference spectrum database created with well-characterized strains cultured in the same condition (18–48 h cultivation).

Our results indicate that TMP-SMX still has a good activity against *Nocardia* species isolated in Japan. TMP-SMX-resistant isolates were found among *N. otitidiscaviarum*, *N. cyriacigeorgica* and *N. mexicana*. The mechanism of resistance to TMP-SMX is being studied mainly in clinically important bacteria such as *Escherichia coli* and *Salmonella* species. It has been reported that acquisition of plasmid-mediated resistance genes (*sul* and *dfr*) and chromosomal gene mutations in the *dhps* and *dhfr* genes coding for the target enzymes dihydropteroate synthase (DHPS) and dihydrofolate reductase (DHFR), respectively, is the major resistance mechanisms^22–24^ in such bacteria. To the best of our knowledge, the mechanism of TMP-SMX resistance in *Nocardia* species has not yet been clarified, although a recent study reported that the acquisition of plasmid-mediated resistance genes is involved in high resistance as in general bacteria. Valdezate et al. investigated 76 high-level TMP-SMX-resistant *Nocardia* isolates (≥ 32/608 μg/ml) isolated in Spain between 2007 and 2013, and found that these isolates possessed either one or multiple plasmid-mediated sulfonamide- and/or trimethoprim-resistant genes (*sul1,* 93.4%; *sul2,* 78.9%; and *dfrA*, 14.7%)^25^. In the present study, we could not find such a strain in the five TMP-SMX-resistant isolates. The MICs of these TMP-SMX-resistant isolates ranged from 4/76 to 8/152 μg/ml, and there were no high-level TMP-SMX-resistant *Nocardia* isolates. These results suggest that low level resistance around the MIC breakpoints may occur with different resistant mechanisms. Mehta et al. conducted an in vitro experimental evolution study to adapt susceptible clinical isolates of *N. cyriacigeorgica* and *N. nova* to the treatment of choice, TMP-SMX. They found that chromosomal gene mutations were seen within genes encoding DHFR, DHPS and a homolog (*folp2*) of the gene encoding DHPS in experimental de novo resistant strains^26^. While their study did not include sequence data of clinically TMP-SMX-resistant *Nocardia* strains, they suggested that chromosomal gene mutations may be implicated in low-level TMP-SMX resistance identical to that of other bacterial species, such as *E. coli*^27^.

On the other hand, it is known that TMP-SMX therapy is strongly associated with the emergence of thymidine-dependent small colony variants (SCVs) in *Staphylococcus* species^28^. Underlying mutations have been identified for thymidine-dependent SCVs in *S. aureus*, and mutations of the *thyA* gene have been shown to be responsible for the SCV phenotype^29^. The SCVs have also been found in some clinically important bacteria such as *Stenotrophomonas maltophilia*, *P. aeruginosa*, *E. coli*, *Salmonella* species, and *Enterococcus* species^30,31^. The SCV phenotype is characterized by small colony size, slow growth on agar media compared to wild-type isolates, and the inability to generate in vitro susceptibility results under standard conditions, as defined by CLSI^30^. Unfortunately, to date there have been no reports on SCVs in *Nocardia* species. Mehta et al. reported that a point mutation was observed at 16 bp upstream of *thyA*, which is an operon with the DHFR gene (*folA*), in experimental de novo TMP-SMX-resistant *Nocardia* strains, although they did not investigate the relationship between the mutation and SCV phenotype^26^. *Nocardia* infections are not uncommon in immunosuppressed patients receiving TMP-SMX for prophylaxis^1,32^; therefore, the existence of SCVs in *Nocardia* species cannot be denied. Further, TMP-SMX is frequently used not only for prophylaxis, but also for long-term treatment over 6 months^1^, so it is necessary to elucidate the resistance mechanisms, including chromosomal gene mutations and SCVs, and to develop an accurate detection method for TMP-SMX-resistant strains.

Tigecycline is the first in a new class of antimicrobials, a member of the glycylcyclines, and is an analogue of minocycline with additional properties that negate most mechanisms, mediating resistance to tetracyclines^33^. In vitro testing has revealed that tigecycline is active against Gram-positive cocci, including *Enterococcus* species, *S. aureus* and *Streptococcus pneumoniae*, and many species of multi-drug-resistant Gram-negative bacteria^33^. Lai et al. investigated 151 clinical isolates of *Nocardia* species, and reported that tigecycline had a low MIC_90_ (1 μg/ml), and that MIC values were ≤ 8 μg/ml against all of the tested isolates, suggesting the potential clinical application of tigecycline for the treatment of nocardiosis^34^. In the present study, tigecycline had a low MIC distribution only for *N. brasiliensis*, *N. otitidiscaviarum* and some clinically unusual *Nocardia* species. Some researchers have reported that *N. farcinica* complex, *N. nova* complex and *N. transvalensis* complex isolates were less susceptible to tigecycline than *N. abscessus*, *N. brasiliensis*, or *N. otitidiscaviarum*^35,36^. Further studies are needed to demonstrate the clinical role of tigecycline in the management of nocardiosis.

To our knowledge, the present study is the first to have evaluated the activity of arbekacin against a diverse range of *Nocardia* species. Arbekacin is a broad-spectrum aminoglycoside licensed for systemic use in Japan and Korea, where it is usually used to treat methicillin-resistant *S. aureus* infections^37–39^. Matsumoto et al. reported that arbekacin is stable against aminoglycoside-inactivating enzymes such as (3′) aminoglycoside-phosphotransferase, (4′) aminoglycoside-adenyltransferase (AAD), or AAD (2″) and has a weak affinity for (6′-IV) aminoglycoside-acetyltransferase^40,41^. Therefore, arbekacin has antimicrobial activity against Gram-positive and -negative pathogens, including strains resistant to gentamicin, tobramycin, and amikacin^40,42^. In this study, arbekacin was four-fold more active than amikacin, and showed low MIC values even against *N. transvalensis* complex, which is reported to be resistant to all aminoglycosides^43^. These results indicate that arbekacin has a good potential to be a concomitant antibiotic for empirical therapy or therapy for serious nocardiosis infections.

In conclusion, the current study demonstrated that MALDI-TOF MS is a quick, easy and reliable method for the species/complex identification of *Nocardia* species. Accurate identification by MALDI-TOF MS and antimicrobial susceptibility profiles together can help earlier implementation of appropriate antimicrobial treatment and improvement of patient prognosis.

## Supplementary information

Supplementary Figure 1.

Supplementary Table 2.

## References

[1] Wilson JW (2012). Nocardiosis; Updates and clinical overview. Mayo Clin. Proc..

[2] Hemmerabach-Miller M, Stout JE, Woodworth MH, Cox GM, Saullo JL (2018). *Nocardia* infections in the transplanted host. Transpl. Infect. Dis..

[3] Margalit I (2021). How do I manage nocardiosis?. Clin. Microbiol. Infect..

[4] Conville PS (2012). Multisite reproducibility of the broth microdilution method for susceptibility testing of *Nocardia* species. J. Clin. Microbiol..

[5] Brown-Elliott BA, Brown JM, Conville PS, Wallace RJ (2006). Clinical and laboratory features of the *Nocardia* spp. based on current molecular taxonomy. Clin. Microbiol. Rev..

[6] Conville PS, Brown-Elliott BA, Witebsky FG, Carroll KC (2019). *Nocardia*, *Rhodococcus*, *Gordonia*, *Actinomadura*, *Streptomyces*, and other aerobic actinomycetes. Mannual of clinical microbiology.

[7] Blosser SJ (2016). Multicenter matrix-assisted laser desorption ionization-time of flight mass spectrometry study for identification of clinically relevant *Nocardia* spp. J. Clin. Microbiol..

[8] Khot PD, Bird BA, Durrant RJ, Fisher MA (2015). Identification of *Nocardia* species by matrix-assisted laser desorption ionization-time of flight mass spectrometry. J. Clin. Microbiol..

[9] Segawa S (2015). Identification of *Nocardia* species using matrix-assisted laser desorption/ionization-time-of-flight mass spectrometry. Clin. Proteomics..

[10] Nakamura I, Omori N, Umeda A, Ohkusu K, Matsumoto T (2015). First case report of fatal sepsis due to *Campylobacter upsaliensis*. J. Clin. Microbiol..

[11] Conville PS, Brown-Elliott BA, Smith T, Zelazny AM (2018). The complexities of *Nocardia* taxonomy and identification. J. Clin. Microbiol..

[12] Clinical and Laboratory Standards Institute. Interpretive criteria for identification of bacteria and fungi by DNA target sequencing; approved guideline. In *Clinical and Laboratory Standards Institute document MM18-A* (Clinical and Laboratory Standards Institute, 2008)

[13] Clinical and Laboratory Standards Institute. Susceptibility testing of mycobacteria, nocardiae, and other aerobic actinomycetes; approved standard, 2nd ed. In *Clinical and Laboratory Standards Institute document M24-A2* (Clinical and Laboratory Standards Institute, 2011) 31339680

[14] Kozak GK, Boerlin P, Janecko N, Reid-Smith RJ, Jardine C (2009). Antimicrobial resistance in *Escherichia coli* isolates from swine and wild small mammals in the proximity of swine farms and in natural environments in Ontario, Canada. Appl. Environ. Microbiol..

[15] Argudin MA (2011). Virulence and resistance determinants of German *Staphylococcus aureus* ST398 isolates from nonhuman sources. Appl. Environ. Microbiol..

[16] Saitou N, Nei M (1987). The neighbor-joining method: A new method for reconstructing phylogenetic trees. Mol. Biol. Evol..

[17] Tamura K, Nei M, Kumar S (2004). Prospects for inferring very large phylogenies by using the neighbor-joining method. Proceedings of the National Academy of Sciences (USA).

[18] Kumar S, Stecher G, Li M, Knyaz C, Tamura K (2018). MEGA X: Molecular evolutionary genetics analysis across computing platforms. Mol. Biol. Evol..

[19] Hsueh PR (2014). Bruker Biotyper matrix-assisted laser desorption ionization-time of flight mass spectrometry system for identification of *Nocardia*, *Rhodococcus*, *Kocuria*, *Gordonia*, *Tsukamurella*, and *Listeria* species. J. Clin. Microbiol..

[20] Verroken A (2010). Evaluation of matrix-assisted laser desorption ionization-time of flight mass spectrometry for identification of *Nocardia* species. J. Clin. Microbiol..

[21] Toyokawa M (2013). Reliable and reproducible method for rapid identification of *Nocardia* species by matrix-assisted laser desorption/ionization time-of-flight mass spectrometry. Rinsho Biseibutshu Jinsoku Shindan Kenkyukai Shi.

[22] Huovinen P, Sundstrőm L, Swedberg G, Skőld O (1995). Trimethoprim and sulfonamide resistance. Antimicrob. Agents Chemother..

[23] Perreten V, Boerlin P (2003). A new sulfonamide resistance gene (*sul3*) in *Escherichia coli* is widespread in the pig population of Switzerland. Antimicrob. Agents Chemother..

[24] Antunes P, Machado J, Sousa JC, Peixe L (2005). Dissemination of sulfonamide resistance genes (*sul1*, *sul2*, and *sul3*) in Portuguese *Salmonella enterica* strains and relation with integrons. Antimicrob. Agents Chemother..

[25] Valdezate S, Garrido N, Carrasco G, Villalón P, Medina-Pascual MJ, Saéz-Nieto JA (2015). Resistance gene pool to co-trimoxazole in non-susceptible *Nocardia* strains. Front. Microbiol..

[26] Mehta H, Weng J, Prater A, Elworth AL, Han X, Shamoo Y (2018). Pathogenic *Nocardia cyriacigeorgica* and *Nocardia nova* evolve to resist trimethoprim-sulfamethoxazole by both expected and unexpected pathways. Antimocrob. Agents Chemother..

[27] Mehta HH, Shamoo Y (2020). Pathogenic *Nocardia*: A diverse genus of emerging pathogens or just poorly recognized?. PLoS Pathog..

[28] Kahl BC, Becker K, Lőffler B (2016). Clinical significance and pathogenesis of Staphylococcal small colony variants in persistent infections. Clin. Microbiol. Rev..

[29] Chatterjee I (2008). In vitro mutations of thymidylate synthase (encoded by *thyA*) are responsible for thymidine dependency in clinical small-colony variants of *Staphylococcus aureus*. J. Bacteriol..

[30] Anderson SW, Stapp JR, Burns JL, Qin X (2007). Characterization of small-colony variant *Stenotrophomonas maltophilia* isolated from the sputum specimens of five patients with cystic fibrosis. J. Clin. Microbiol..

[31] Johnes BE, Purdy KJ, Tucker NP, Maddocks SE (2015). Phenotypic and genotypic characteristics of small colony variants and their role in chronic infection. Microbiol. Insight.

[32] Coussement J (2016). *Nocardia* infection in solid organ transplant recipients: a multicenter European case-control study. Clin. Infect. Dis..

[33] Pankey GA (2005). Tigecycline. J. Antimicrob. Chemother..

[34] Lai CC (2011). Multicenter study in Taiwan of the in vitro activities of nemonoxacin, tigectcline, doripenem, and other antimicrobial agents against clinical isolates of various *Nocardia* species. Antimicrob. Agents Chemother..

[35] Schlaberg R, Fisher MA, Hanson KE (2014). Susceptibility profiles of *Nocardia* isolates based on current taxonomy. Antimicrob. Agents Chemother..

[36] Lebeaux D (2019). Antibiotic susceptibility testing and species identification of *Nocardia* isolates: a retrospective analysis of data from a French expert laboratory, 2010–2015. Clin. Microbiol. Infect..

[37] Watanabe T (1987). Antibacterial activities of arbekacin, a new aminoglycoside antibiotic, against methicillin-cephem-resistant *Staphylococcus aureus*. Jpn. J. Antibiot..

[38] Matsumoto T (2013). Clinical efficacy and safety of arbekacin sulfate in patients with MRSA sepsis or pneumonia: A multi-institutional study. J. Infect. Chemother..

[39] Hwang JH, Lee JH, Moon MK, Kim JS, Won KS, Lee CS (2013). The efficacy and safety of arbekacin and vancomycin for the treatment in skin and tissue MRSA infection: preliminary study. Infect. Chemother..

[40] Matsumoto T (2014). Arbekacin: another novel agent for treating infections due to methicillin-resistant *Staphylococcus aureus* and multidrug-resistant Gram-negative pathogens. Clin. Pharmacol..

[41] Matsumoto T, Yamamoto H (1988). The enzymatic mechanisms of resistant to aminoglycoside antibiotics in methicillin-cephem-resistant *Staphylococcus aureus*. Jpn. J. Antibiot..

[42] Sader HS, Rhomberg PR, Farrell DJ, Jones RN (2015). Arbekacin activity against contemporary clinical bacteria isolated from patients hospitalized with pneumonia. Antimicrob. Agents Chemother..

[43] Conville PS, Brown JM, Steigerwalt AG, Brown-Elliott BA, Witebsky FG (2008). *Nocardia wallacei* sp. Nov. and *Nocardia blacklockiae* sp. Nov., human pathogens and members of the “*Nocardia transvalensis* complex”. J. Clin. Microbiol..

