# Supplementary figures and images for: Identification and antimicrobial susceptibility profiles of Nocardia species clinically isolated in Japan
Source: Sci Rep. 2021 Aug 18;11:16742. doi: 10.1038/s41598-021-95870-2 (PMC8373947; doi:10.1038/s41598-021-95870-2)

## Slide 1
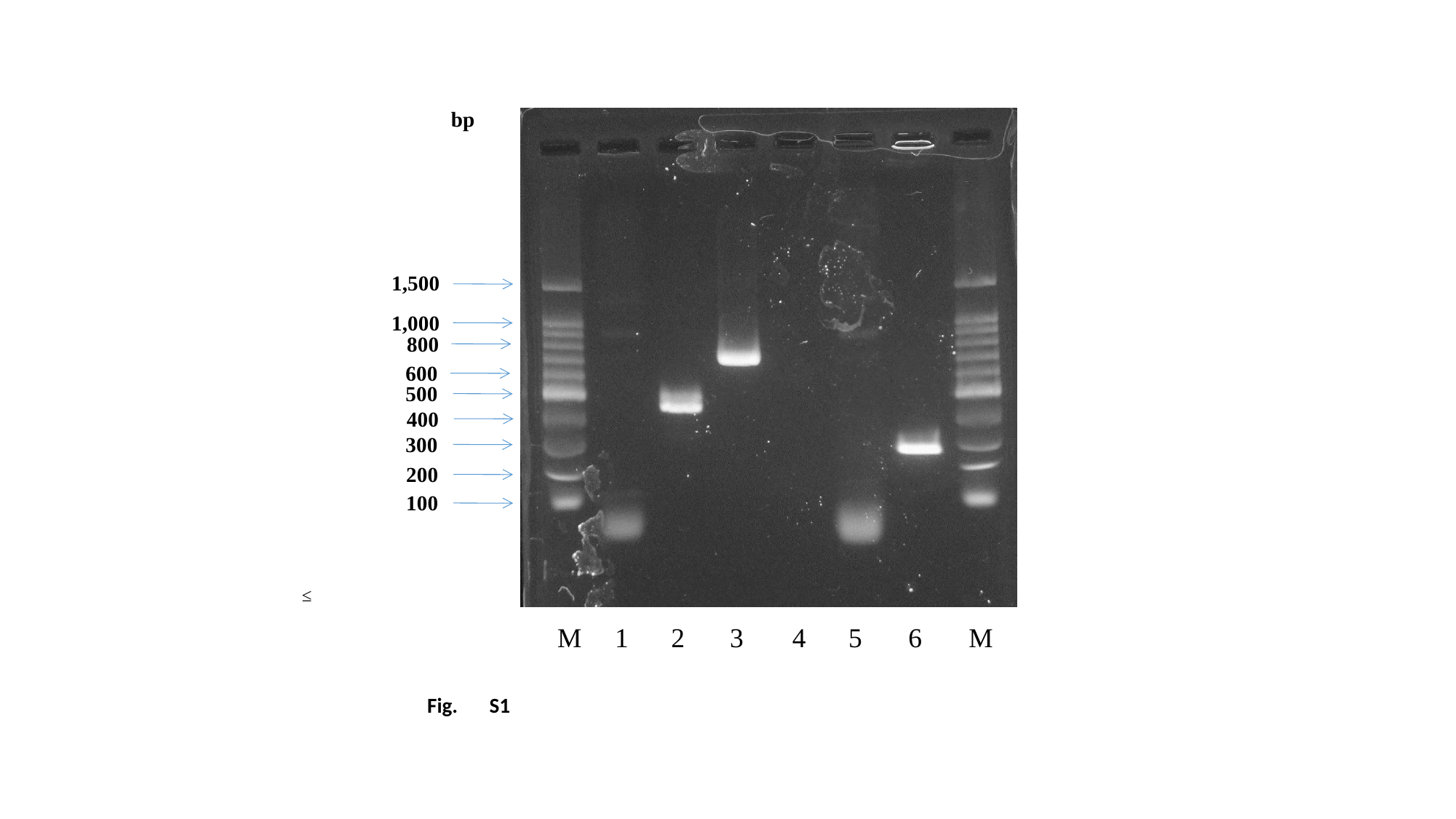

bp
1,500
1,000
800
600
500
400
300
200
100
≤
M
1
2
3
4
5
6
M
Fig.　S1

Supplement: Supplementary file 1 — Supplementary Figure 1. [file 41598_2021_95870_MOESM1_ESM.pptx]
