# Supplementary material for: Identification and antimicrobial susceptibility profiles of Nocardia species clinically isolated in Japan
Source: Sci Rep. 2021 Aug 18;11:16742. doi: 10.1038/s41598-021-95870-2 (PMC8373947; doi:10.1038/s41598-021-95870-2)
Supplement: Supplementary file 2 — Supplementary Table 2. [file 41598_2021_95870_MOESM2_ESM.docx]

| Group | Gene | Primer sequence 5’-3’ | Product size (bp) | Reference |
| --- | --- | --- | --- | --- |
| Sulfonamide resistance | *sul1* | CGGCGTGGGCTACCTGAACG | 433 | (14) |
|  |  | GCCGATCGCGTGAAGTTCCG |  |  |
|  | *sul2* | CGGCATCGTCAACATAACCT | 721 | (14) |
|  |  | TGTGCGGATGAAGTCAGCTC |  |  |
| Trimethoprim resistance | *dfrA* | CACTTGTAATGGCACGGAAA | 270 | (15) |
|  |  | CGAATGTGTATGGTGGAAAG |  |  |

Table S1.
